# Earache and Chloroform Vapor

**Published:** 1880-07

**Authors:** 


### EARACHE AND CHLOROFORM VAPOR.

Dr. Morgan states that he has often promptly relieved the distressing earache of children by filling the bowl of a common new clay pipe with cotton wool, upon which he dropped a few drops of chloroform, and inserted the stem carefully into the external canal, and adjusting his lips over the bowl, blew through the pipe, forcing the chloroform vapor upon the membrana tympani.—*National Medical Review.*

